# *Dictyostelium polycephalum* Infection of Human Cornea

**DOI:** 10.3201/eid1610.100717

**Published:** 2010-10

**Authors:** Ashok Kumar Reddy, Praveen Kumar Balne, Prashant Garg, Virender Singh Sangwan, Madhusmita Das, Pravin V. Krishna, Bhupesh Bagga, Geeta K. Vemuganti

**Affiliations:** Author affiliation: L.V. Prasad Eye Institute, Hyderabad, India

**Keywords:** Dictyostelium polycephalum, ameba, slime mold, human infection, cornea, keratitis, letter

**To the Editor:** Although *Dictyostelium* spp. are used for studying signal transduction, cytoskeletal functions, endocytosis, and molecular pathogenesis of infectious and other diseases (*1*), human or animal infections caused by this organism have not been reported. We report a case of keratitis caused by *Dictyostelium polycephalum* in an immunocompetent person.

A 35-year-old man sought treatment for redness, pain, and watering in the left eye of 11 days’ duration. He had no history of ocular injury or surgery. At the time of his medical visit, he was using ophthalmic solutions of 5% natamycin sulfate, 0.5% moxifloxacin hydrochloride, and 0.3% gentamicin sulfate, each instilled every hour, and 1% atropine sulfate, 3×/d.

The vision in his right eye and results of a clinical examination were within normal limits. His left eye visual acuity was expressed as the ability to count fingers at 1 m. The eyelids were edematous and the conjunctivae were congested. The cornea showed a large central epithelial defect with underlying stromal infiltrate and Descemet folds. The surrounding cornea had a mild cellular reaction. The anterior chamber was deep, and the pupil was round, regular, and dilated. Iris and lens details could not be distinguished because of corneal haze. We obtained corneal scrapings, and the material was subjected to a detailed microbiologic analysis (*2*).

Microscopic examination showed double-walled spherical cysts in potassium hydroxide with calcofluor white stain, Gram stain (Figure, panels A, B), and Giemsa stain. On the basis of this finding, a presumptive diagnosis of *Acanthamoeba* keratitis was made. The patient was advised to use 0.02% polyhexamethylene biguanide and 0.02% chlorhexidine eye drops every half hour and 1% atropine eye drops 3×/d and was asked to return for a follow-up visit the next day. However, the patient did not return and could not be located. After 48 hours’ of incubation, a nonnutrient agar plate showed growth of double-walled, spherical cysts ≈6–7 µm in diameter that had different morphologic features than those of *Acanthamoeba* spp. cysts.

**Figure:**
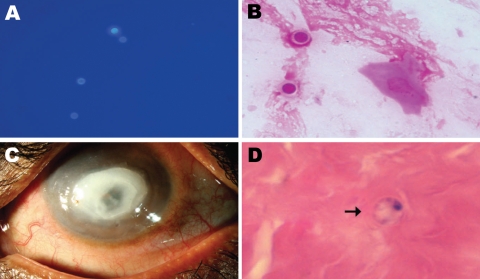
A) Spherical cysts of *Dictyostelium* spp. in potassium hydroxide (calcoflour white stain; original magnification ×40) preparation. B) Spherical double wall cysts of *Dictyostelium* spp. (Gram stain; original magnification ×100). C) Cornea of the patient’s left eye, showing a ring-shaped central infiltrate and central thinning. D) Corneal button showing *Dictyostelium* spp. cysts (arrow; hematoxylin and eosin stain; original magnification ×100).

To identify the organism, we extracted DNA from the growth on nonnutrient agar and subjected it to PCR specific for *Acanthamoeba* spp (*3*); results were negative. The extracted DNA was then subjected to 18S rDNA PCR for free-living amebas as described by Tsvetkova et al. (*4*). A PCR product ≈800 bp was obtained and subjected to bidirectional sequencing with fluorescent-labeled dideoxy nucleotide terminators by using ABI 3130 XI automated sequencer in accordance with the manufacturer’s instructions (PE Applied Biosystems, Foster City, CA, USA).

The Mega BLAST search program (www.ncbi.nlm.nih.gov/blast/megablast.shtml) of GenBank identified the sequence as *D. polycephalum* (99% similarity with AM168056). We deposited the sequence of our isolate in GenBank (accession no. GU562439). The organism showed cytotoxicity after in vitro inoculation of a rabbit corneal epithelial cell line.

The patient sought treatment 4 months after his initial visit. The left eye visual acuity was now expressed as the ability to see hand movements near the face. Slit-lamp examination showed lid edema and conjunctival congestion. The cornea showed a ring-shaped infiltrate, central thinning, surrounding corneal edema, and pigments on the endothelium (Figure, panel C); these findings were identical to the clinical picture of *Acanthamoeba* keratitis. Repeat corneal scrapings showed organisms of same morphologic features seen on the first visit by microscopy and culture. Organisms were reidentified as *D. polycephalum* by sequencing.

Because we were not aware of any drug treatment recommendations for infection by this organism, and the disease was advanced, surgical treatment was advised. Deep anterior lamellar keratoplasty was performed after 2 days. Histopathologic examination of the corneal button showed spherical cysts in mid stroma and inflammatory infiltrates (Figure, panel D). At the last follow-up (3 months after surgery), the corneal graft was clear with no evidence of infection.

Members of the genus *Dictyostelium* (social amebas or cellular slime molds) are divided into 4 high-level taxa with several species on the basis of DNA phylogeny (*5*). The life cycle of *Dictyostelium* spp. consists of an ameboid vegetative phase, a cyst phase, and a plantlike fruiting phase (*6*). *D. polycephalum* is ancestral and show different characteristics than other species of *Dictyostelium* (*5**,**7**,**8*). In culture, it grows at a temperature of 34°C–35°C, which is higher than that for other species of *Dictyostelium* (*8*). Most myxamoebae aggregate to form sporocarps; however, some may round up in individual cells to form microcysts (*8*).The *D. polycephalum* isolated from our patient grew at 36°C on nonnutrient agar with an *Escherichia coli* overlay. The myxamoebae were seen after 24 hours, and the amebae had transformed into microcysts after 48 hours of incubation. However, on further incubation for 3 weeks at 36°C, no sporocarp formed.

Although we could identify the microorganism, the source of infection is unknown. Because the patient was a manual laborer, he could have become infected with the organism from contaminated water or soil. The clinical picture for keratitis caused by *D. polycephalum* was indistinguishable from that caused by *Acanthamoeba* spp. However, careful attention to cyst morphology in clinical samples and culture enabled us to identify this organism**.**
